# Cellular activation of hypothalamic hypocretin/orexin neurons facilitates short-term spatial memory in mice

**DOI:** 10.1016/j.nlm.2016.10.005

**Published:** 2016-12

**Authors:** Teemu Aitta-aho, Elpiniki Pappa, Denis Burdakov, John Apergis-Schoute

**Affiliations:** a Department of Pharmacology, University of Cambridge, Cambridge CB2 1PD, UK; b Division of Neurophysiology, MRC National Institute for Medical Research, London NW7 1AA, UK; c MRC Centre for Developmental Neurobiology, King’s College London, London WC2R 2LS, UK; d Department of Neuroscience, Psychology & Behaviour, University of Leicester, Leicester LE1 7RH, UK

**Keywords:** Hypothalamus, Recognition memory, Spontaneous alternation, Orexin

## Abstract

The hypothalamic hypocretin/orexin (HO) system holds a central role in the regulation of several physiological functions critical for food-seeking behavior including mnemonic processes for effective foraging behavior. It is unclear however whether physiological increases in HO neuronal activity can support such processes. Using a designer rM3Ds receptor activation approach increasing HO neuronal activity resulted in improved short-term memory for novel locations. When tested on a non-spatial novelty object recognition task no significant difference was detected between groups indicating that hypothalamic HO neuronal activation can selectively facilitate short-term spatial memory for potentially supporting memory for locations during active exploration.

## Highlights

• Activation of HO neurons increases the expression of the activity marker c-Fos.
• HO activation improves spatial short-term memory for novel locations.
• Similar increases in HO activity had no effect on object recognition memory.

## 1 Introduction

Foraging requires the coordination of higher-order neural systems that lead to a heightened sensory awareness, effective decision-making, and mnemonic functions for remembering objects and locations previously experienced. The engagement of these circuits must ultimately be derived from primary brain circuits that respond to energy deficiencies for increasing arousal for engaging food seeking behavior and achieving energy homeostasis. The hypocretin/orexin (HO) system holds a central role in the regulation of activity for exploration as it has been shown to be a key system in promoting food intake, arousal, reward, and, importantly, food anticipatory activity (Carter et al., 2009, De Lecea, 2010, Kotz, 2006, Sakurai et al., 1998, Zhang et al., 2013). Although the cell bodies of HO neurons are confined to the lateral hypothalamus (LH), they project extensively to the entire neuroaxis (Tsunematsu & Yamanaka, 2012) supporting a role for this system in coordinating multiple brain systems for effective foraging behavior, including extra-hypothalamic memory-related regions (Nambu et al., 1999, Peyron et al., 1998). In agreement with the above neuroanatomy, pharmaco-behavioral experiments have demonstrated a functional role for the HO system in hippocampal and amygdala-dependent memory processing (Jaeger et al., 2002, Palotai et al., 2014, Sears et al., 2013, Telegdy and Adamik, 2002). Electrophysiology studies however have revealed that HO neurons can functionally excite and inhibit neurons independent of HO release/receptor activation (Apergis-Schoute et al., 2015, Schöne et al., 2014). Despite these studies linking HO receptor activation to mnemonic functions it is unclear whether physiological increases in HO activity alone can support such processes.

The aim of this study was to test the effects of HO neuronal activation on short-term memory for locations and objects. To do so, a genetic tool – the designer receptors exclusively activated by designer drugs (DREADDs) – was implemented in an HO-cre^+^ transgenic mouse line in order to directly excite HO neurons during task engagement. Due to potential confounding effects of food reward as a positive reinforcer the memory tasks in the present study were ones that were “unbaited”, (without food), namely the spontaneously alternating T-maze (Deacon and Rawlins, 2006, Lalonde, 2002) and the spontaneous object recognition task (Bussey et al., 1999, Leger et al., 2013, Winters and Bussey, 2005) both of which are thought to be motivated by the instinct to explore novel locations and objects.

## 2 Materials and Methods

### 2.1 Animal subjects

All animal procedures were performed in accordance with the United Kingdom Animals (Scientific Procedures) Act of 1986. Male HO-cre^+^ mice were bred in heterozygous: wild-type (wt) breeding pairs with C57BL/6J mice and were genotyped using PCR from ear notch biopsy. Heterozygous HO-cre^+^ and their wt littermates (Matsuki et al., 2009, Sasaki et al., 2011) were selected and used for all the behavioral tests after the age of 9 weeks. The animals were group housed (2–4 animals per cage), under temperature- and humidity-control conditions, on a 12-h light/dark reversed cycle (7 a.m. lights off). Water and food were available ad libitum during all the experimental procedures.

### 2.2 Stereotaxic operations and viral gene transfer

A Cre-inducible AAV_5_-hSyn-DIO-rM3Ds-mCherry or AAV_5_-hSyn-DIO-mCherry viral construct (titer 4 ∗ 10^12^ genome copies/mL, University of North Caroline, Gene Therapy Center, NC, USA) was bilaterally injected into the LH of male HO-cre^+^ and wt littermate mice. Ten-week old HO-cre^+^ mice and their wt littermates were anesthetized with a mixture of isoflurane (5% induction, 1–2% maintenance) and oxygen (flow rate 0.8–1.0 L/min), and placed into a stereotactic frame. A small bilateral craniotomy was performed for injections of viral constructs (150 nL) at 0.1 μL/min (antero-posterior −1.35 mm from bregma, laterally ±0.95 mm from midline and −5.4 mm, −5.2 mm and −5.0 mm from the skull surface). Postoperatively, mice received meloxicam (1 mg/kg. s.c., Boehringer Ingelheim Ltd. Bracknell, UK) and behavioral tests started after a 5-week period to allow for sufficient protein expression. All surgeries were done using aseptic surgical techniques.

### 2.3 Electrophysiology

Coronal slices were made >9 weeks post-injection and recordings were made as in our previous study (Apergis-Schoute et al., 2015). Data were analyzed with Minianalysis (Synaptosoft), Igor and Adobe Illustrator/Photoshop. Whole-cell recordings were performed at 35 °C using an EPC-10 amplifier and Patch-Master software (HEKA Elektronik).

### 2.4 Chemicals and solutions

ACSF was gassed with 95% O_2_ and 5% CO_2_, and contained the following (mM): NaCl 125, NaHCO_3_ 25, KCl 3, NaH_2_PO_4_ 1.25, CaCl_2_ 1/2 (cutting/recording), MgCl_2_ 6/1 (cutting/recording), sodium pyruvate 3 and glucose 25/5 (cutting/recording). Pipettes were filled with (in mM): potassium gluconate 135, NaCl 7, Hepes 10, Na_2_-ATP 2, Na-GTP 0.3, and MgCl_2_ 2; pH was adjusted to 7.3 with KOH. Clozapine-N-oxide (CNO, Sequoia Research Products Ltd, Pangbourne, UK) was freshly dissolved in sterile saline (0.9% NaCl) with 0.5% DMSO and administered by intraperitoneal injection (i.p.) 30 min prior to behavioral tasks. All chemicals were from Sigma, Tocris, or Bachem.

### 2.5 Transcardial perfusion and tissue preparation

Mice were anaesthetized with 500 mg/kg of sodium pentobarbital and were tested for absence of paw pinch response. Mice were perfused with 50 mL of phosphate buffer solution (PBS) and subsequently with 50 mL of 4% paraformaldehyde at pH 7.4. Brains were removed and post-fixed in 4% paraformaldehyde overnight and then kept in 30% sucrose for cryoprotection. Coronal sections of 30 μm were cut by a sliding microtome.

### 2.6 Immunohistochemistry

The primary and secondary antibodies used were as follows: primary; goat anti-c-Fos (1:800, Santa Cruz Biotechnology, sc-52-G, Santa Cruz, CA, USA) and rabbit anti-orx-A (1:1000, Phoenix Pharmaceuticals, H-003-30, Burlingame, CA, USA). Secondary; donkey Biotin-SP-conjugated anti-goat (1:500, Jackson ImmunoResearch, 705-065-003, Jennersville, PA, USA) and AlexaFluor 488-conjugated goat anti-rabbit (1:1000, Abcam, ab150077, Cambridge, UK). All antibodies were diluted in blocking solution (0.3% TritonX-100, 10% donkey serum, 1% BSA in PBS). Sections were incubated at room temperature and rinsed 3 × 5 min in PBS, then incubated for 1 h in 0.3% H_2_O_2_ in PBS and subsequently in blocking solution, for 1 h and incubated in anti-c-Fos primary antibody, overnight. The next day sections were incubated in biotinylated anti-goat secondary antibody for 1 h, followed by 5-min incubation in peroxidase-conjugated avidin/biotin complex, ABC solution (Vector laboratories, Peterborough, UK) and in DAB substrate. Sections were then washed, mounted onto slides and cover-slipped. Images were captured with a Zeiss fluorescence microscope and analyzed by ImageJ software.

### 2.7 Behavioral assays

#### 2.7.1 Open field test

An open field test was used to assess potential differences in locomotor activity and novelty-induced anxiety levels between the groups (Hall & Ballachey, 1932). Mice were injected with CNO (1 mg/kg, i.p.) and 30 min later were allowed to move freely into an cylindrical open field for a total test time of 30 min, under low light intensity (<50 lx) conditions. The arena of 36 cm in diameter was used to test mouse locomotor activity and anxiety-like behavior in an open field and was divided into three 12-cm wide circular zones (outer, middle, and center zone) (Fig.2A). Video recording was used to track the open field behavior, by measuring the total distance moved and time spent in each zone. Subsequently, off-line video analysis (EthoVision version 11, Noldus Information Technology, Wageningen, The Netherlands) was conducted to quantify behavior.

#### 2.7.2 Spontaneous alternation T-maze test

Spontaneous alternation in a T-maze was used to assess working memory abilities, based on the innate tendency of mice (and rats) to alternate their choice of a novel goal arm based on the recall of their initial choice (Olton & Papas, 1979). The T-maze used was made of black painted wood and consisted of one central arm (starting area) and two goal-arms, with dimensions 30 cm × 10 cm each and walls 20 cm in height, as well as a removable central divider extending 5 cm into the start arm. The protocol used was based on the work of Deacon and Rawlins (2006). Vehicle or CNO (1 mg/kg, i.p.) was administered in a pseudo-randomized way, 30 min prior to the test, and mice were placed in the start arm with the central divider in place so that when entering either of the goal arms the mice were not able to see the other one. After the mice entered either of the arms, a black painted wooden wall placed in front of the chosen arm’s entrance, to allow them to explore the arm for 30 s. After a delay of 0 or 5 min, mice were returned to the start arm, facing away from the goal arms, with the central divider and wall removed. A schematic representation of the T-maze apparatus and the behavioral assay used is provided in Fig.3A. Mice were tested once daily at a constant time and injected with CNO or vehicle on alternate days. Trials on which the mice did not complete the task were excluded. All the mice completed 7–11 trials per condition.

#### 2.7.3 Spontaneous novel object recognition test

Object recognition memory was tested in a Y-shaped maze (walls 30 cm high, each arm 16 cm long and 8 cm wide, made of white acrylic plastic) in low light level (<50 lx) and recorded using video camera for subsequent off-line analysis. The mice were habituated to the empty maze in two consecutive daily trials that lasted 5 min each. The spontaneous object recognition testing consisted of two phases. First, mice were injected with CNO (1 mg/kg, i.p.) 30 min before the trial and then placed in the maze and allowed to explore two identical objects for 5 min. Thereafter, each mouse was returned to its home cage. For the choice trial, 30 min post-sample phase each mouse was returned to the maze for 5 min and presented with one sample object and a novel object. The side of the novel object was counterbalanced. A schematic representation of the spontaneous object recognition task and the apparatus used is provided in Fig.3B. Exploration was defined as the direct approach of a mouse with an object at a distance closer than 2 cm, but sitting on or chewing an object was not counted as exploration. The data were presented as discrimination ratio calculated as the time spent exploring the novel object minus the time spent exploring the sample object divided by the total exploration time.

## 3 Results

### 3.1 HO neuronal excitation through rM3Ds receptor-activation

**Fig. 1:**
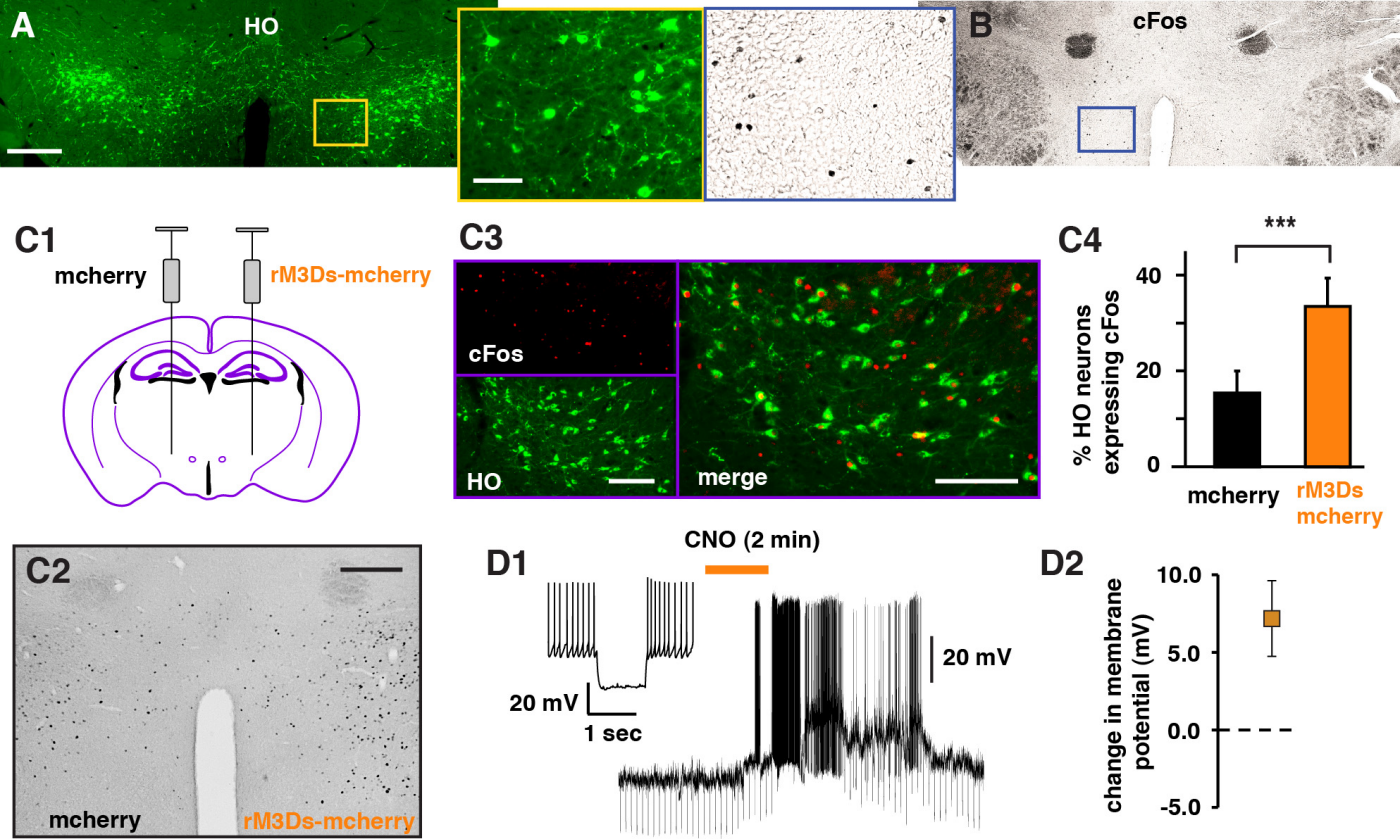
HO neuronal excitation through rM3Ds receptor-activation. HO (A) and cFos (B) immunostaining in the lateral hypothalamus. C1. Schematic representation of the unilateral viral injections at the lateral hypothalamic area of HO-cre^+^ mice. One hemisphere was injected with AAV_5_-hSyn-DIO-rm3Ds-mCherry viral constructs and the other with AAV_5_-hSyn-DIO-mCherry viral construct, which served as control. C. The rM3Ds-expressing hemisphere showed increased cFos expression (C2) compared to control mCherry-only hemisphere. HO-cFos dual immunolabeling (C3) revealed a significant increase in cFos-expressing HO neurons in rm3Ds-expressing hemisphere compared to mCherry-only expressing hemisphere (C4). D. Whole cell recordings from labeled HO neurons expressing rM3Ds DREADDs. Bath application of 10 μM CNO depolarized recorded neurons (D1) on average by 7.2 ± 2.4 mV (D2). Scale bars: (A, B) far left/right, 0.5 mm; middle, 0.1 mm; C2, 0.25 mm C3; 0.20 mm.

Using a cre-recombinase dependent approach for expressing rM3Ds in HO neurons of HO-cre^+^ transgenic mice we measured HO activation in response to the rM3Ds-specific ligand clozapine-N-oxide (CNO). We injected HO-cre^+^ animals with rM3Ds-mCherry and mCherry-only genetic constructs in contralateral hemispheres of individual mice (Fig.1C1). Following a 5-week viral-expression period animals received intraperitoneal injections of 1 mg/kg CNO and were perfused 2 h later for immunohistochemical analysis of cFos, a molecular marker of neuronal activity (Dragunow and Faull, 1989, Morgan and Curran, 1991). CNO injections markedly increased the expression of cFos in HO neurons in the rM3Ds-mCherry expressing hemisphere compared to mCherry-only expressing hemisphere (33.4 ± 5.9%, 15.2 ± 4.7%; n = 4, Student’s Paired *T*-Test, p = 0.002) (Fig.1C). We next prepared acute slices from rM3Ds-mcherry expressing brains and performed whole-cell recordings from labeled neurons (Fig.1D). These neurons were mostly spontaneously active *in vitro* and the injected hyperpolarizing current responses were consistent with previous electrophysiological signatures of HO neurons (Fig.1D2). In the presence of both glutamate and GABA receptor antagonists (in μM, CNQX 20, AP5 100, CGP 10, gabazine 10) we measured the membrane potential changes in response to bath-application of CNO (10 μM) (Fig.1D1). CNO application depolarized neurons by 7.2 ± 2.4 mV (n = 6) (Fig.1D2) supporting our cFos results that CNO can effectively activate rM3Ds-expressing HO neurons.

### 3.2 Activation of orexin neurons does not alter locomotor activity or novelty-induced anxiety

**Fig. 2:**
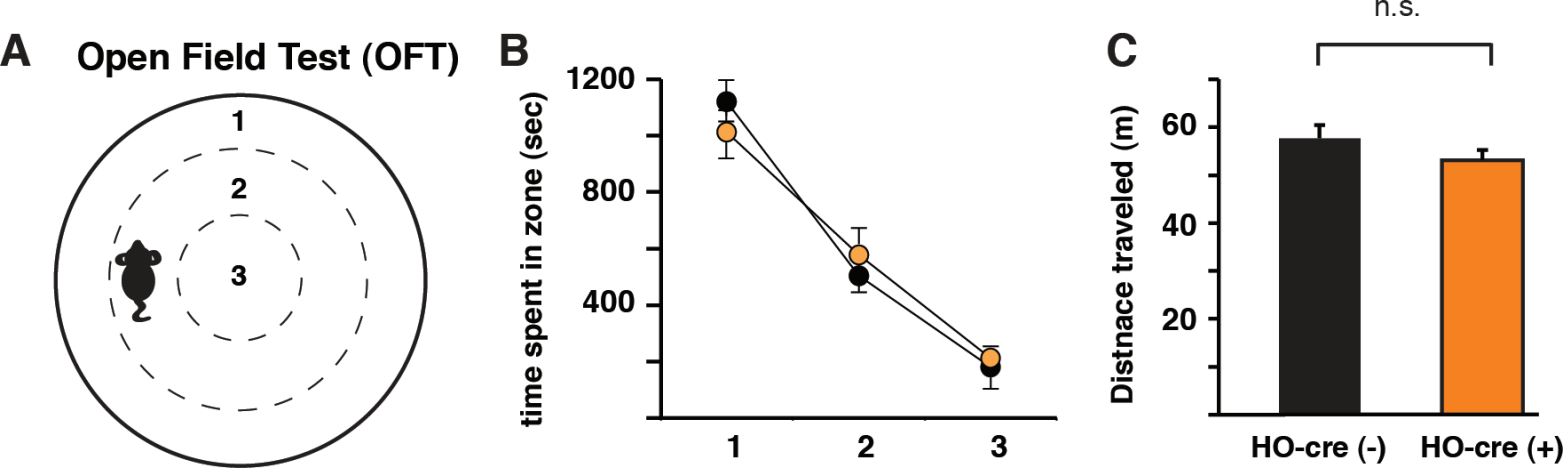
Activation of HO neurons does not alter the general locomotor activity and novelty-induced anxiety. A: Schematic representation of the cylindrical open field arena used in the 36 cm in diameter open field test, divided into three 12-cm wide circular zones (outer, middle, and center zone). In the open field test both groups received CNO injections (1 mg/kg, i.p.) 30 min prior to testing. B: AAV_5_-hSyn-DIO-rM3Ds-mCherry injected HO-cre^+^ and wt mice moved equal distance during a 20 min free exploration period under low light intensity (<50 lx) conditions. C: Open field was divided into three zones and time spent in each zone per genotype was analyzed off-line with EthoVision (version 11, Noldus Information Technology, Wageningen, The Netherlands).

The HO system has been shown to increase locomotor activity (Inutsuka et al., 2014, Kotz, 2006) and heighten fear-related states (Johnson et al., 2010, Lungwitz et al., 2012, Palotai et al., 2014). To determine whether related behavioral changes are induced shortly after HO activation we injected bilaterally-expressing M3Ds-mCherry HO-cre^+^ mice and wildtype (wt) littermate controls with CNO (1 mg/kg) and tested all animals 30 min later in an open field test (OFT) (Fig. 2) during their active phase. HO-cre^+^ mice and wt littermates were injected bilaterally with rM3Ds-mCherry viral constructs, only the latter of which expressed the DREADD protein. In the OFT, animals prefer to position themselves in close proximity to the wall as this reduces susceptibility to predators, an innate behavior known as thigmotaxis. CNO-injected HO-cre^+^ (n = 9) and wt mice (n = 8) moved an equal distance (wt 57.66 ± 2.70 m, HO-Cre^+^ 53.15 ± 2.09 m; Student’s Unpaired *T*-Test, p = 0.20) (Fig.2C). A two-way repeated ANOVA revealed a significant zone effect, with mice spending more time in the outer zone (zone effect, F(2, 32) = 77.28; p < 0.001), and no significant genotype effect on the time spent in each zone (F(1, 16) = 1.95; p = 0.18) (Fig.2B). Taken together, these data indicate that under the current experimental conditions the activation of hypothalamic HO neurons did not result in overt changes in locomotor activity or anxiety/arousal levels.

### 3.3 Investigating the impact of HO neurons on short-term location and spontaneous object recognition memory

**Fig. 3:**
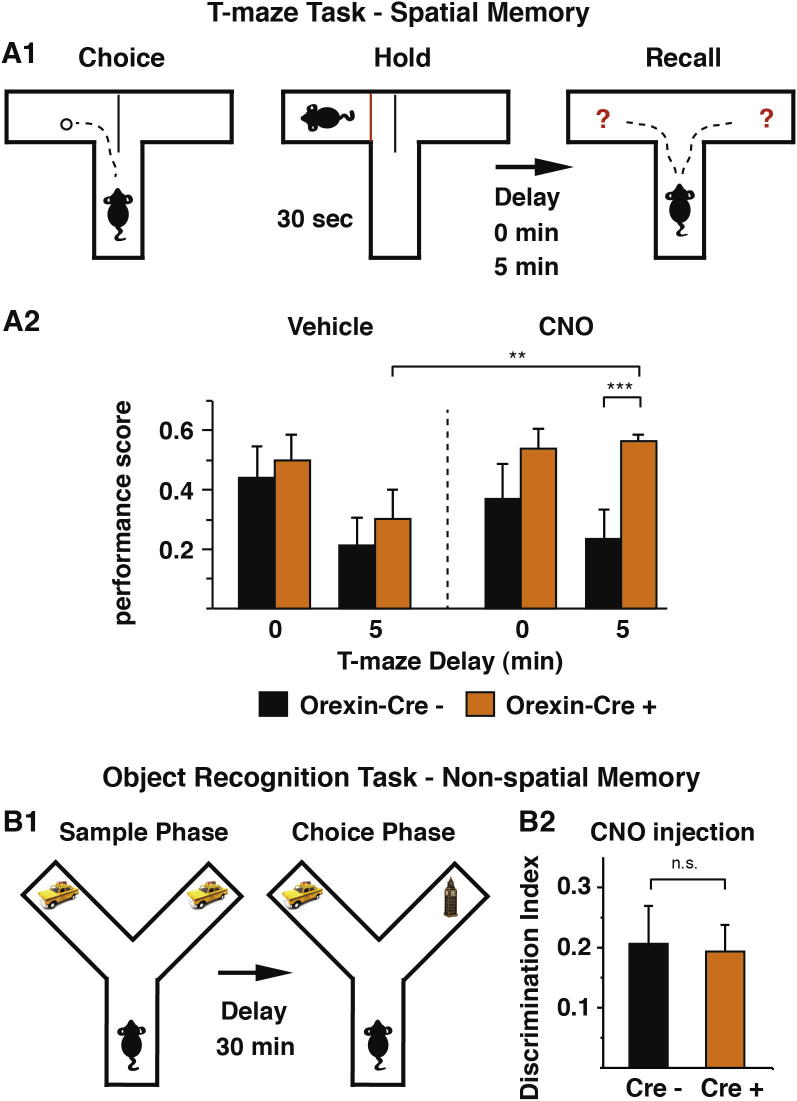
Chemogenetic activation of HO neurons on short-term memory. A1. Schematic representation of the T-maze experimental task (Deacon and Rawlins, 2006). A2. T-maze performance expressed as a normalized performance score, upon vehicle or CNO injections, with zero (0) or five (5) min delay between the choice and recall phase. B1. Schematic representation of the spontaneous object recognition task and the apparatus used. B2. Summary of results from the spontaneous object recognition task. The data were presented as discrimination ratio that was calculated as the time spent exploring the novel object minus the time spent exploring the sample object divided by the total exploration time. ^∗∗^ p = 0.01, ^∗∗∗^ p = 0.001.

To determine whether neuronal activation of the HO circuit can support mnemonic functions bilaterally-expressing M3Ds-mCherry HO-cre^+^ mice and wt littermate controls were tested on a delayed spontaneous alternation T-maze test (Fig.3A1), a task designed for studying spatial short-term working memory (Deacon & Rawlins, 2006). A 5 min delay between encoding and retrieval was chosen for taxing short-term memory. All animals were subject in a randomized fashion to 10 trials of 4 conditions; vehicle injection (veh) – no test delay, CNO-injection (CNO) – no test delay, veh – 5 min test delay, CNO – 5 min test delay. We calculated the percent above chance performance on all 4 conditions for each animal and used a square root transformation of these values to stabilize the variance and normalize the distribution. A mixed two-way ANOVA with repeated measures revealed a statistically significant difference in performance between HO-cre^+^ (n = 10) and wt animals (n = 7) (F(1, 16) = 5.132, p = 0.038), Bonferroni corrected for multiple comparisons with an effect of delay (F(1, 16) = 5.742, p = 0.029) and an interaction of injection ∗ delay (F(1, 16) = 6.168, p = 0.024). As can be seen in Fig.3A2, post hoc between group *t*-tests confirmed that HO-cre^+^ animals performed significantly better than wt mice only on the 5 min delay with CNO (t(16) = −3.868, p = 0.001) and post hoc within group tests per delay demonstrated that HO-cre^+^ mice performed significantly better at the 5 min delay when injected with CNO compared to vehicle (t(9) = 3.01, p = 0.01). We next analyzed arm entry latency and found no differences between the groups (HO-cre^+^ 9.9 ± 2.1 ms, wt 7.8 ± 1.4; Student’s Unpaired *T*-Test, p = 0.47) indicating that, similar to the open field results, these manipulations do not result in significant changes in locomotor activity. These results indicate that HO activation through rM3Ds stimulation facilitates memory in a spatially-based short-term memory task. Notably, no statistical differences were seen between saline injected HO-cre^+^ and wt groups indicating no difference in baseline responding between HO-cre^+^ and wt mice.

We next asked whether the HO system is also involved in supporting non-spatial memory. Here, we tested animals using the non-food baited object recognition task (Fig.3B1) (Leger et al., 2013). Animals were injected with CNO and 30 min later were allowed to explore two identical objects for 5 min (sampling phase) and thereafter were returned to their home cage. The choice phase was conducted after a short (30 min) delay from the sample phase, during which the mice were placed back to the maze and presented with one of the sample objects and a novel object. When tested on a spontaneous object novelty recognition task CNO-injected rM3Ds-expressing HO-cre^+^ mice (n = 9) did not show altered memory for a familiar object compared to CNO-injected wt mice (n = 7) (30 min test, wt 0.19 ± 0.05 Discrimination Index (DI), HO-cre^+^ 0.21 ± 0.06 DI; Student’s Unpaired *T*-Test, p = 0.56) (Fig.3B2).

## 4 Discussion

The ubiquitous projection pattern of hypothalamic HO neurons is suggestive of HO playing an influential role in the coordination of multiple brain regions linked to foraging behavior including ones important for mnemonic processing. Our results indicate that global increases in HO activity improve performance in delayed spontaneous alternations when short-term memory is taxed with a 5 min delay between encoding and retrieval and further implicates the HO system in supporting memory functions during active exploration. Disruption of HO transmission via HO receptor antagonists or selective HO lesions has been shown to adversely affect initial learning (Akbari et al., 2006, Sears et al., 2013, Soya et al., 2013, Telegdy and Adamik, 2002), consolidation (Akbari et al., 2006), or retrieval (Akbari et al., 2006, Telegdy and Adamik, 2002) depending on the region involved. In light of data indicating that CNO can activate DREADDs for up to a few hours (Krashes et al., 2011), HO neuronal activity in our study was likely elevated throughout all behavioral experiments. As a result the current investigation cannot differentiate between a selective HO involvement in memory encoding, maintenance (5 min delay), or recall. The general rM3Ds-mediated increase in HO activity likely resembles the *in vivo* condition since HO activity is elevated during wakefulness (Lee et al., 2005, Mileykovskiy et al., 2005), mostly during bouts of active exploration (Kotz, 2006, Mileykovskiy et al., 2005).

A recent study using a similar methodology (DREADD-mediated HO activation with 1 mg/kg CNO) however has shown that HO circuit activation resulted in an increase in locomotor activity (Inutsuka et al., 2014). In contrast to our experimental design, this experiment was performed during the light phase when locomotor and HO activity is minimal suggesting that the HO-mediated increases in locomotor activity documented in this study may have more to do with an increase in arousal/wakefulness than in a general increase in locomotor activity. This interpretation is supported by a subsequent experiment where they show that disruptions in the HO system have no effect of normal light-dark locomotor activity (Inutsuka et al., 2014). Another possibility is that the degree of activation was insufficient to affect locomotor activity since in our study the HO system was activated by a Gs- and not a Gq-linked DREADD, the latter of which is known to excite neurons to a larger degree (Pei, Rogan, Yan, & Roth, 2008). These unique Gs and Gq signaling mechanisms may result in dissimilar increases in the firing frequencies of HO neurons resulting in differences in glutamate and HO release and corresponding postsynaptic impact (Schöne et al., 2014).

In contrast to the improvement in delay spontaneous alternation memory, activating the HO circuit during a spontaneous novel object recognition task had no impact on memory. Previous work has implemented the social recognition memory task, an alternative non-spatial memory task, to investigate the involvement of the HO system on short-term recognition memory. In this study disrupting the HO system resulted in animals spending an equal amount of time interacting with familiar and unfamiliar mice indicating that social memory was negatively affected by disruptions in the HO system. The possible discrepancy in HO contributions to non-spatial recognition memory between our results and those of Yang et al. (2013) may be due to a number of factors including the methodology involved (HO disruption or activation, respectively), the fact that these processes are encoded by different circuits, or both. In contrast to Yang et al. (2013), where they disrupted HO function and mostly performed their behavioral studies during the animals least active light-phase, we chose to investigate how activating the HO system during the animals’ active phase impacts non-spatial memory thereby avoiding potential confounds in regards to decreased levels of arousal/wakefulness associated with HO dysfunction that can profoundly affect memory. We therefore believe that differences in methodology make it difficult to directly compare the results between the studies. In addition, sensory processing for conspecifics is inherently different than for novel objects and therefore might engage different pathways that are impacted differently by the HO system.

Our results indicate that activating the HO system can contribute to short-term memory for novel locations but the HO target regions mediating this facilitation remains unresolved. Candidate pathways include memory-related brain regions or neuromodulator systems both of which have been shown to be under HO control. For example, hippocampus-directed HO receptor antagonism has been shown to disrupt spatial memory (Akbari et al., 2008, Akbari et al., 2006) despite the fact that HO neurons sparsely innervate the hippocampus (Chen et al., 1999, Peyron et al., 1998). Extensive work has shown that the medial prefrontal cortex (mPFC) plays an important role not only in spatial memory (Brown and Bowman, 2002, Curtis and D’Esposito, 2004) but also in the maintenance of sensory representations when stimuli are absent (Horst and Laubach, 2009, Larsen and Divac, 1978, Spellman et al., 2015). Through mPFC innervation HO inputs can excite prefrontal projection neurons through direct (Li et al., 2010, Xia et al., 2005) and indirect mechanisms (Lambe and Aghajanian, 2003, Lambe et al., 2005) the latter of which is correlated with improvements in attention. The mPFC has been shown to be important in delayed spontaneous alternation but so have a number of other brain regions including the hippocampus, the dorsomedial striatum, and various thalamic nuclei (reviewed in Lalonde, 2002). Neuromodulatory cell populations have also been implicated in delayed alternation memory (Bubser and Schmidt, 1990, Lalonde, 2002, Talley et al., 2000), many of which are regulated by HO inputs (Peyron et al., 1998). These HO projections can potentially act to increase the activity of various arousal-related cell populations (Peyron et al., 1998, Sakurai and Mieda, 2011, Sears et al., 2013, Vittoz and Berridge, 2006) that in turn can support mnenomic functions. Our results indicate that activating the HO system can facilitate delayed alternation memory but the mechanism and pathways involved are currently unresolved.

Pharmacological and ablation studies have demonstrated that spatial short-term memory is dependent on a number of different modulator systems acting in various memory-related brain regions (Lalonde, 2002). Our results demonstrate that cellular increases in HO activity that resemble the *in vivo* condition (Mileykovskiy et al., 2005) can facilitate short-term memory for locations, further linking the hypothalamic HO system to spatial memory. Furthermore, our data provide a basis for determining the causal links between HO circuits to memory by directly manipulating HO neuronal activity. Through the divergent innervation of multiple brain regions involved in memory processing, our data indicate that the hypothalamic HO system can potentially support foraging behavior through the online engagement of mnemonic functions necessary for building spatial maps for effective navigation.
